# Symbiont-mediated feminization imposes unavoidable host fitness costs

**DOI:** 10.3389/fmicb.2026.1798411

**Published:** 2026-04-30

**Authors:** Jordyn D. Robinson, Dylan T. Thorp, Jeremy Van Cleve, Jennifer A. White

**Affiliations:** 1 Department of Entomology, Agricultural Science Center North, University of Kentucky, Lexington, KY, United States; 2 Department of Biology, T.H. Morgan Building, University of Kentucky, Lexington, KY, United States

**Keywords:** bacterial endosymbionts, feminization, reproductive manipulation, sex determination, symbiosis, *Wolbachia*

## Abstract

Maternally inherited bacterial endosymbionts such as *Wolbachia* are common in arthropods. Some serve as reproductive manipulators, favoring the production of infected females in host populations despite possible fitness costs to the host. One such manipulation is feminization, in which the symbiont turns genetic males into functional females. To date, all described cases of feminization occur in host systems that are either female heterogametic (ZW-female/ZZ-male) or where females are diploid and males are haploid for sex chromosomes (XX-female/X0-male). Here we test potential fitness costs associated with feminization in the spider *Mermessus fradeorum* (Linyphiidae), which has a type of XX/X0 sex determination. In addition to a feminizing *Wolbachia*, this spider can be co-infected with up to four additional maternally-inherited bacterial endosymbionts. Using a series of increasingly speciose symbiont co-infections, including three containing the feminizing *Wolbachia*, we measured female fecundity and the proportion of developed versus undeveloped offspring. We found that fitness costs were associated only with the feminizing *Wolbachia*, but not with any of the other symbionts. Eggmasses infected with this *Wolbachia* had 16% fewer eggs, and 20% of those eggs failed to develop, compared to only 4% failure in eggmasses from other symbiotypes. We hypothesize that the reduced egg viability results from the production of inviable 00 zygotes by feminized X0 individuals, which can provision X chromosomes to only half of their eggs. These results suggest that fitness costs may be an unavoidable consequence of feminization in hosts with an XX/X0 sex determination system, potentially limiting the distribution of this reproductive manipulation phenotype.

## 1 Introduction

Arthropods are commonly infected with maternally inherited bacterial endosymbionts that persist and spread in host populations, even when they impose fitness costs upon their hosts (Oliver et al., 2006; Moran et al., 2008; Doremus and Oliver, 2017). These microbes are host-restricted with rare opportunity for horizontal (contagious) spread, meaning that the fitness of the microbial lineage is directly tied to the fitness of the infected host. Consequently, such endosymbionts are under selective pressure to minimize costs to their host, thus promoting host survival and reproduction and allowing the microbial lineage to persist and transmit. Indeed, many maternally-transmitted endosymbionts are obligately or conditionally beneficial for their hosts (Kaltenpoth et al., 2025). Endosymbionts can also manipulate the host’s reproduction, often to favor the production of infected female hosts (Werren et al., 2008; Doremus and Hunter, 2020). These manipulations function to increase the relative number of hosts that can transmit the bacteria, allowing spread within the population even if the microbes decrease host fitness (Turelli, 1994; Engelstädter et al., 2004; Shropshire et al., 2020). This symbiont-driven reproductive manipulation can occur in multiple forms, including male-killing, induction of parthenogenesis, cytoplasmic incompatibility, and feminization of genetic males into functional phenotypic females (Duron et al., 2008; Shropshire et al., 2020). Such manipulating symbionts are likewise under selective pressure to minimize fitness costs to their hosts and have been documented to evolve reduced costs over time (Weeks et al., 2007). However, some host manipulations impose direct and unavoidable host fitness costs. Male killing, in particular, trades off a loss of male offspring with increased fitness for their surviving infected sisters (Hurst and Jiggins, 2000; Doremus and Hunter, 2020).

**Figure 1:**
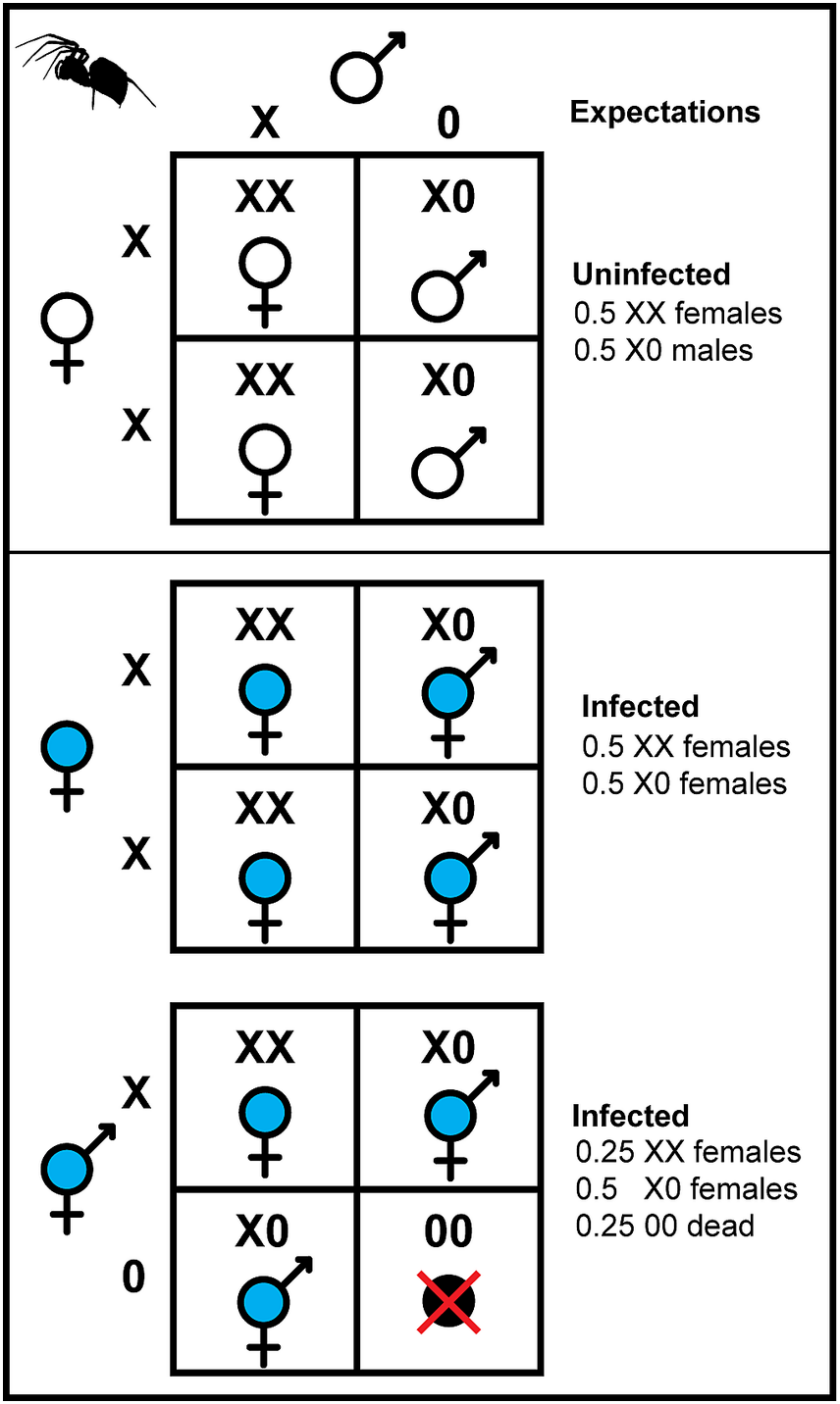
Expected karyotype distribution and offspring sex ratio for uninfected (white) and infected (blue) females with an XX/X0 sex determination system. Uninfected females produce half uninfected XX females and half uninfected X0 males. Infected females may be either natural XX females or feminized X0 individuals that are also functional females. Infected XX females produce half infected XX females and half infected X0 females. Infected X0 females produce one fourth infected XX females, one half infected X0 females, and one fourth 00 offspring, which are presumed to be inviable. This expectation extends to more complicated diploid/haploid sex chromosome karyotypes such as the X_1_X_1_X_2_X_2_/X_1_X_2_ system exhibited by spiders such as *Mermessus fradeorum*.

Here, we explore the idea that, similar to male-killing symbionts, at least some feminizing symbionts also impose unavoidable fitness costs on their hosts. Feminization is thought to be the least common form of manipulation, but it affects a wide array of arthropods including isopods (Rigaud et al., 1999), spiders (Curry et al., 2015), butterflies (Hiroki et al., 2004; Kageyama et al., 2012), and leafhoppers (Negri et al., 2006). All described cases of feminization occur in arthropods with either female heterogametic (ZW female/ZZ male) or XX female/X0 male sex determination systems. The mechanisms by which symbionts impose feminization appear to be diverse (Negri et al., 2006; Kageyama et al., 2012; Herran et al., 2020) and may impose fitness costs on the host (Rigaud et al., 1999). In X0 sex determination systems, females are diploid XX and males are haploid X0 for the sex chromosome. If symbionts feminize genetic males to functional females and these feminized individuals retain their X0 karyotype, half of their eggs would not receive an X chromosome during meiosis (Figure 1). Eggs that lack an X would form viable (and feminized) zygotes if they unite with a sperm that contains an X, but if the sperm also lacks an X chromosome, then the resulting 00 zygote would likely be inviable. With normal segregation, we’d therefore expect that feminized X0 individuals would experience a 25% reduction in viable progeny relative to their XX counterparts.

**Figure 2:**
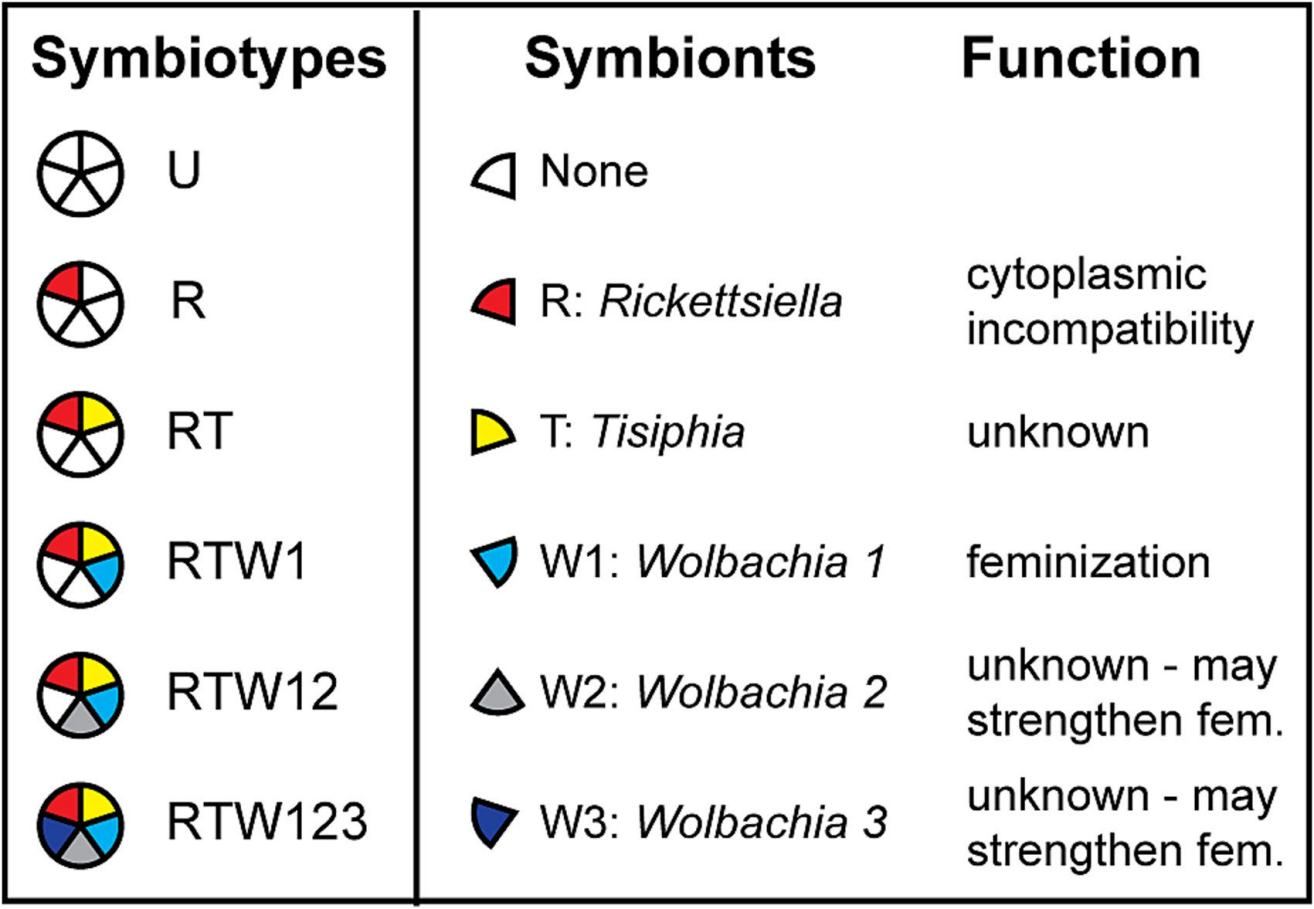
Spiders infected with different combinations of symbionts (symbiotypes) used in this study. Each symbiont is represented by a different color and symbol as indicated in column 2. Where known, symbiont function is indicated in column 3.

We tested this hypothesis with a linyphiid spider, *Mermessus fradeorum*. This species exhibits a type of XX/X0 karyotype, X_1_X_1_X_2_X_2_ female/X_1_X_2_ male (Curry et al., 2015). For simplicity, we will use the XX/X0 notation even in this more complicated instance. This spider is infected with varying combinations of five maternally inherited bacterial endosymbionts: virtually all local individuals are infected with a *Rickettsiella* (abbreviated as R), and a subset of typically 30–50% of individuals are additionally co-infected with some combination of a *Tisiphia* (T), and three strains of *Wolbachia* (W1, W2, and W3; Rosenwald et al., 2020, Rosenwald, 2020; Figure 2). One of the *Wolbachia* strains, dubbed *Wolbachia 1*, is present in ~20% of local spiders, frequently in combination with all four other symbionts, and is necessary for feminization. We have previously shown that all tested symbiont combinations that included *Wolbachia 1* had strongly female-biased offspring, and all combinations that lacked *Wolbachia 1* had even sex ratios (Mackevicius-Dubickaja et al., 2025). Of the other symbionts, *Rickettsiella* causes a different form of reproductive sabotage, cytoplasmic incompatibility (Rosenwald et al., 2020), which results in inviable undeveloped zygotes in crosses between *Rickettsiella*-infected males and females that lack *Rickettsiella*. The other symbionts have unknown function, although some may intensify feminization (Mackevicius-Dubickaja et al., 2025). We contrasted egg development amongst spiders with different combinations of symbionts (hereby referred to as “symbiotypes”) that did versus did not include *Wolbachia 1* to look for evidence of inviable 00 eggs associated with the feminizing symbiont. The presence of other symbionts alongside *Wolbachia 1* also allowed us to address whether co-infection *per se* imposes additional fitness costs upon the host (Rigaud et al., 1999; Oliver et al., 2006; Doremus and Oliver, 2017; Rock et al., 2018). We hypothesize that symbiotypes containing the feminizing *Wolbachia 1* will produce a greater proportion of undeveloped eggs relative to spiders of non-feminized symbiotypes due to these inviable 00 pairings.

## 2 Materials and methods

Laboratory spider lines were originally collected from alfalfa fields in Fayette County, Kentucky (38.127 N, 84.508 W). We maintained spiders in growth chambers at a constant 21 °C on a diet of *Sinella curviseta* collembola as spiderlings and *Drosophila melanogaster* fruit flies as adults (Proctor et al., 2024). We initially screened each lineage for symbiotype with a 16S-based microbiome protocol (Kozich et al., 2013; Rosenwald et al., 2020), then periodically verified infection via diagnostic polymerase chain reactions (PCRs; see below). To obtain uninfected lines, we cured infected spiders using antibiotics (Curry et al., 2015) and maintained lineages for multiple generations before experimental use. Wild spiders are infected with varying combinations of up to five symbionts, with *Rickettsiella*-only and 5-fold infected spiders being the most common; uninfected spiders are extremely rare in local wild populations. Antibiotic and heat treatments have failed to generate spider lineages that stably maintain other symbionts without a co-infection with *Rickettsiella* (J. White, unpublished data). Our approach therefore was to compare a series of increasingly speciose symbiotypes (Figure 2), allowing us to evaluate the effect of adding each symbiont to the consortium, as well as compare multiple symbiotypes that do or do not include the feminizing *Wolbachia 1*.

We compared offspring development across six different symbiotypes (Figure 2). We reared spiders from each symbiotype in individual cups in the same growth chamber at 21 °C under the same feeding schedule for 9–10 weeks until they reached sexual maturity. We randomly selected 30 females from each symbiotype for experimental use; for those carrying *Wolbachia 1,* the selected spiders constituted an unknown mixture of genetic XX females and feminized X0 individuals, whereas the other symbiotypes were all genetic females. All females from all symbiotypes were mated to uninfected males, to prevent cytoplasmic incompatibility in the uninfected spiders and maintain uniformity among the crosses. We allowed each female to lay one egg mass, starved her for 5 days to clear gut contents, and then preserved her in 95% ethanol for molecular analysis.

We opened each eggmass 12 days after it was laid. At this time, viable spiderlings had hatched, but remained in a teneral state (Foelix, 2011). Under undisturbed conditions, these spiderlings would remain together in the eggmass for a couple more days, emerging together after an additional molt and sclerotization. We used this timing because sclerotized spiderlings within the eggmass are prone to cannibalization and would have precluded accurate counts of undeveloped eggs. We counted the number of undeveloped eggs and developed spiderlings for each egg mass. To verify that undeveloped eggs would not have developed given more time, we retained unhatched eggs in 1.5 mL tubes for three additional days but never saw signs of further development. Any spiders that did not mate initially (*n* = 11, 6.1%) or that had an eggmass with 0% development rate (*n* = 6, 3.3%) were deemed infertile and were excluded from the dataset.

We validated symbiont infection in all experimental spiders using diagnostic PCR. We extracted DNA using Qiagen DNeasy Blood and Tissue kits according to the manufacturer’s protocols, followed by diagnostic PCRs to determine infection status. *Rickettsiella* (recA primers; Proctor et al., 2024), and *Tisiphia* (Ricklong primers; Curry et al., 2015) were evaluated for all specimens. We used a general test for *Wolbachia* (*wsp* primers; Baldo et al., 2006) on samples not expected to be infected with *Wolbachia* and screened for the individual *Wolbachia* strains (primers wsp1, wsp2, and wsp3; Mackevicius-Dubickaja et al., 2026) on samples expected to be infected with *Wolbachia*. Finally, we validated extraction quality using arthropod COI (lco1490 and hco2198 primers; Folmer et al., 1994) on samples that were negative for all symbionts. In the case that a sample’s symbiotype was not as expected (*n* = 1, 0.6% of the overall set), we removed it from the sample set.

Qualitatively, we considered the observed development rate in the feminizing symbiotypes relative to the expected development rate if feminized X0 individuals produced ¼ inviable 00 eggs. Because XX and X0 females are morphologically indistinguishable, we built a mathematical model via recursion equations to determine the expected fraction of XX versus X0 females in the population, assuming the population is in equilibrium (Supplementary material). These calculations assumed perfect symbiont transmission (Mackevicius-Dubickaja et al., 2026, in revision) and high but imperfect feminization of X0 individuals. The latter is important, because feminization failure means X0 individuals become males rather than females, which changes the expected proportion of XX and X0 individuals among the females. We used the observed adult sex ratios of the broods that produced the mothers in this experiment to estimate feminization failure and then separately calculate the expected proportion of XX and X0 females for each of the three symbiotypes bearing *Wolbachia 1* (Supplementary material). We used this expected fraction of XX and X0 females to further calculate the expected proportion of undeveloped eggs in the feminizing symbiotypes, assuming X0 mothers produce 25% inviable 00 eggs and XX mothers do not. To account for background sources of egg mortality besides feminization, we used the proportion of undeveloped eggs in the non-feminizing symbiotypes as a baseline, which was incorporated multiplicatively into the expected proportion of undeveloped offspring in the feminized symbiotypes (Supplementary material). We estimated the probability density function for offspring mortality for each feminizing symbiotype based on its observed number of eggs per eggmass using 500,000 random draws per distribution and generated a 95% confidence interval for each estimate (Supplementary material). We then asked whether our observed mean proportion of undeveloped eggs for each feminizing symbiotype fell within its respective calculated confidence interval.

We compared egg viability (developed and undeveloped offspring) among symbiotypes using logistic regression with Williams’ correction for moderate overdispersion (Arc v 1.06; Williams, 1982). We contrasted the symbiotypes that contained *Wolbachia 1* (RTW1, RTW12, RTW123) to those that did not (Uninfected U, R, RT), and also specifically evaluated the effect of *Wolbachia 1* by contrasting the RTW1 to RT symbiotypes. Finally, we compared the total number of eggs across treatments, as a test of more general fitness costs of the various symbionts on host fecundity. We used a general linear model to compare among all symbiotypes, followed by Tukey’s HSD for separation of means (SPSS v 29.0).

## 3 Results

**Figure 3:**
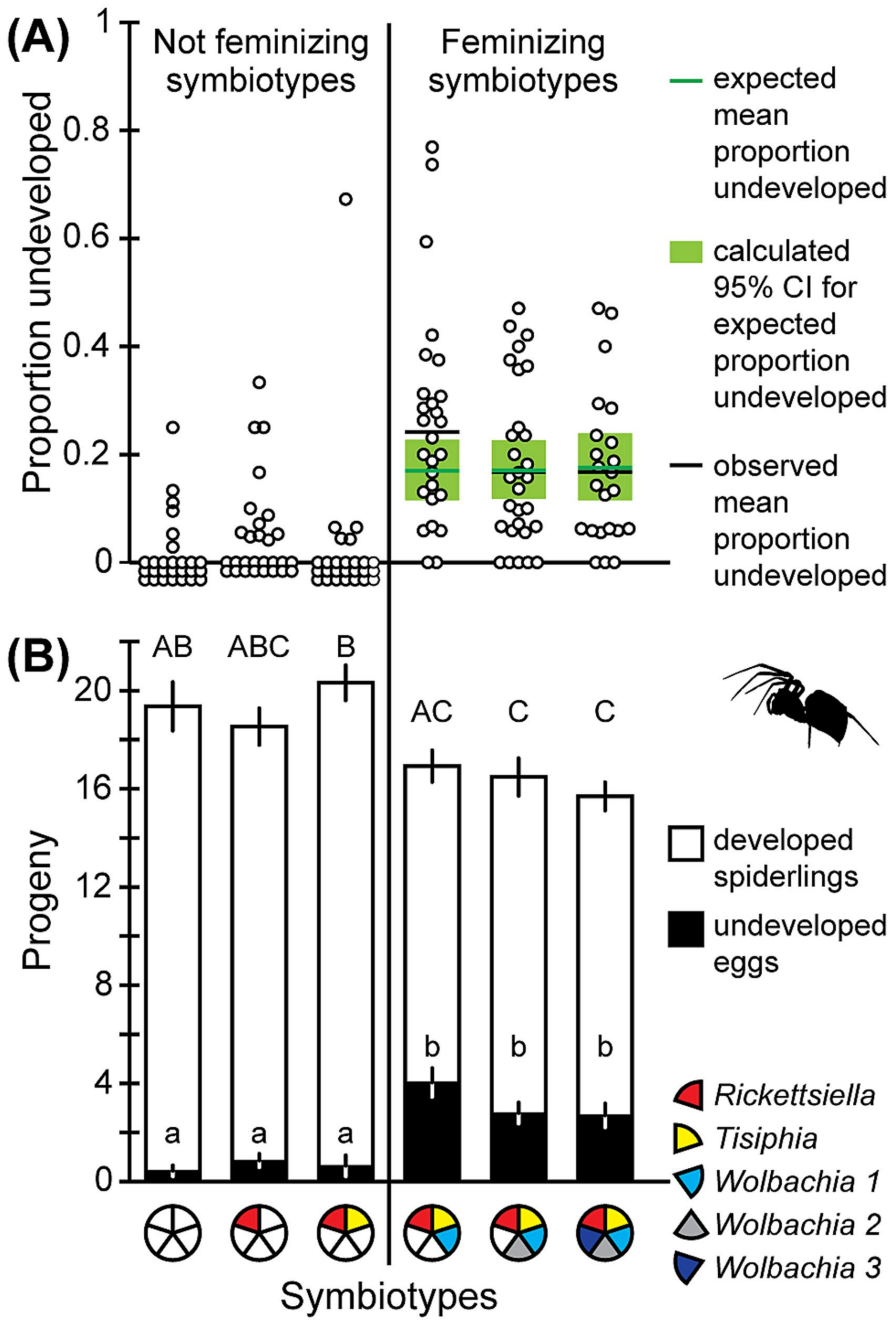
**(A)** Scatterplot of the proportion undeveloped eggs for individual broods of spiders with different symbiotypes. For feminized symbiotypes, the expected mean proportion undeveloped eggs is indicated by the horizontal green line, the calculated 95% confidence interval for this estimate is indicated by the green rectangle, and the observed mean proportion undeveloped eggs is indicated by the horizontal black line. **(B)** Mean ± SE total progeny produced by *Mermessus fradeorum* spiders infected with different combinations of symbionts. Each column is divided into progeny that were developed spiderlings versus undeveloped eggs. Uppercase letters separate total progeny means at *α* = 0.05, lowercase letters separate proportion undeveloped progeny.

Across feminized symbiotypes, we calculated an expected proportion of undeveloped eggs of 0.170 in the RTW1 symbiotype, 0.171 in RTW12, and 0.176 in RTW123 (Figure 3A; Supplementary material). The slightly higher expectation for RTW123 results from better feminization in this group (see Mackevicius-Dubickaja et al., 2025), which inflates the proportion of X0 individuals in the female population, since fewer X0 individuals become males. Corresponding 95% confidence intervals for each estimate range from 0.115 to 0.239 (Figure 3A). When we compared observed proportions of undeveloped eggs to these 95% confidence intervals for each feminized symbiotype (Figure 3A), the observed mean proportion of undeveloped eggs for both RTW12 and RTW123 fell centrally within their 95% confidence interval. The proportion of undeveloped eggs in RTW1, however, was somewhat higher and outside its 95% confidence interval, indicating that this group may have experienced higher than expected proportions of X0 individuals or additional sources of mortality. For RTW12 and RTW123, though, production of inviable 00 eggs provides a reasonable and sufficient explanation for the observed proportion of undeveloped eggs.

The proportion of progeny that did not develop varied among symbiotypes (ΔDeviance = 55.83, d.f. = 5, *p* < 0.001; Figure 3) with ~6.5 × higher odds of undeveloped eggs in the feminized symbiotypes (any containing *Wolbachia 1*) than the non-feminized symbiotypes (ΔDeviance = 52.99, d.f. = 1, *p* < 0.001). Across all feminized symbiotypes, 3.19 ± 0.30 eggs did not hatch per eggmass (a proportion of 0.20 ± 0.02), compared to only 0.67 ± 0.18 eggs per eggmass that did not develop in the unfeminized symbiotypes (a proportion of 0.04 ± 0.01; Figure 3). When we contrasted only the RTW1 symbiotype to the RT symbiotype, thereby isolating the effect the feminizing symbiont *Wolbachia 1* only, we recovered the same pattern (ΔDeviance = 16.6, d.f. = 1, *p* < 0.001).

We additionally found that total offspring production was affected by symbiotype (*F*_5,156_ = 6.065, *p* < 0.001; Figure 3B), with feminized symbiotypes showing a reduction of about three eggs per eggmass (a 16% reduction) relative to unfeminized eggmasses (*t* = −5.152, d.f. = 156, *p* < 0.001). When we compared the means among all symbiotypes, the RTW12 and RTW123 symbiotypes separated from the U and RT symbiotypes, with R and RTW1 intermediate (Figure 3B).

## 4 Discussion

Symbiont-induced feminization imposes clear fitness costs on the host spider, *M. fradeorum*. Spiders bearing the feminizing symbiont *Wolbachia 1* exhibited a markedly higher proportion of undeveloped eggs than spiders lacking this symbiont and had fewer eggs in the eggmass (Figure 3). Given that the proportional development and total progeny did not differ among the three feminized symbiotypes (RTW1, RTW12, and RTW123), nor among the three non-feminized symbiotypes (U, R, and RT), it appears that none of the other symbionts within this host imposed detectable costs, and that the observed fecundity cost is associated specifically with the feminizing symbiont *Wolbachia 1*. Like male-killing, feminization seems to impose an innate cost on the host in this system (Hurst, 1991; Brenninger et al., 2025).

In part, this cost may be a direct consequence of feminized X0 individuals producing eggs that lack X chromosomes. Presuming that X0 mothers produce a mixture of eggs bearing and lacking sex chromosomes, and that our lab populations were composed of an equilibrium mixture of XX and X0 females, we calculated the expected proportion of progeny that would be inviable 00 zygotes. Our observed proportions of undeveloped eggs were largely congruent with this expected proportion, supporting the hypothesis. Kageyama et al. (2017) were able to quantify sex chromosome dosage within a feminized butterfly system, and found that infected females bore only a single Z sex chromosome and lack the usual heterogametic W. These Z0 individuals produced zygotes that were a mixture of haploid and diploid for Z, implying that some female gametes received the Z and some lacked a sex chromosome altogether. When cured of the feminizing symbiont via antibiotics, haploid Z0 offspring were largely inviable, and only diploid male ZZ offspring survived (Kageyama et al., 2017). Our results support a comparable condition in an X0 sex determination system, with the observed proportion of undeveloped eggs consistent with our assumptions of expected levels for 00 offspring. However, the possibility remains that infection with *Wolbachia 1* decreases fitness in other ways that decrease egg development (Weeks et al., 2007; Doremus and Oliver, 2017). Unfortunately, there are no obvious morphological markers to distinguish X0 from XX females in *M. fradeorum*, unlike feminized X0 leafhoppers (Negri et al., 2006). We therefore cannot fully disentangle the effects of infection with *Wolbachia 1* versus effects of X-chromosome haploidy. Further complicating matters, *M. fradeorum* bears two distinct X chromosomes (Curry et al., 2015), as is common in spiders (Král et al., 2011), meaning that females have two copies of both X chromosomes (X_1_X_1_X_2_X_2_) and males have only one of each (X_1_X_2_). Our future efforts to develop X-linked markers for quantifying X-ploidy level of mothers and their gametes will need to account for multiple distinct X chromosomes.

Beyond undeveloped eggs, infection with *Wolbachia 1* also decreased the total number of eggs in the eggmass by about 16%. In general, such reduction in egg allocation in the first eggmass may or may not accurately reflect lifetime fecundity costs (Kleinteich et al., 2015; Dong et al., 2025). Under laboratory conditions, an average *M. fradeorum* is capable of laying more than 10 fertile eggmasses in her lifetime, but the first is usually the largest (J. Robinson, unpublished data). It is theoretically possible that *Wolbachia 1* infected spiders could live longer, produce more eggmasses, or larger eggmasses later in life that partially compensate for early life costs. In practice, however, we have observed no such compensatory effects (J. Robinson, unpublished data) and view this reduction in number of eggs as an additional cost associated with hosting the feminizing symbiont. When combined with the reduced hatch rate, the number of developed eggs produced by spiders with the feminizing symbiont was reduced by 1/3 relative to those without *Wolbachia 1*, which likely undercuts the ability to spread in populations, even when this symbiont strongly promotes the production of female offspring (Curry et al., 2015; Mackevicius-Dubickaja et al., 2025). Co-infection with *Wolbachia 2* and *Wolbachia 3* may somewhat mitigate the costs imposed by *Wolbachia 1* (Figure 3A) and also strengthen feminization (Mackevicius-Dubickaja et al., 2025), suggesting that co-infections may be key to the maintenance of *Wolbachia 1* in *M. fradeorum* populations.

Our results suggest that, similar to male-killing, symbiont-induced feminization may come with hard-wired fitness costs, at least in hosts with XX/X0 sex determination systems. If the strength of the feminization phenotype exceeds these imposed costs and transmission rates are high, a feminizing symbiont could still persist and spread in a host population, as long as the infected hosts produce more daughters than their uninfected counterparts (Turelli, 1994; Brenninger et al., 2025). Clearly, though, these unavoidable fitness costs undercut the numerical daughter-advantage for infected hosts, which would slow symbiont spread within a population, lower equilibrial infection rates, and increase the odds of extinction due to random demographic events in newly invaded populations (Fisher et al., 2024; Brenninger et al., 2025). Speculatively, concomitant fitness costs may be part of the explanation for why feminizing symbionts are often found at low to moderate frequency in field populations (Rigaud et al., 1999, Negri et al., 2006; Narita et al., 2011; Rosenwald et al., 2020; but see Miyata et al., 2024), and are a less frequently observed symbiotic manipulation of arthropod hosts.

## Data Availability

The original contributions presented in the study are publicly available. This data can be found here: 10.5281/zenodo.19656452.
