# Supplementary material for: Symbiont-mediated feminization imposes unavoidable host fitness costs
Source: Front Microbiol. 2026 Apr 30;17:1798411. doi: 10.3389/fmicb.2026.1798411 (PMC13171762; doi:10.3389/fmicb.2026.1798411)
Supplement: Supplementary file 1 [file Data_Sheet_1.PDF]

Supplementary Material for: "Symbiont-Mediated Feminization Imposes Unavoidable Host Fitness Costs"

1 MATHEMATICAL MODEL OF FEMINIZING SYMBIONT SPREAD

In the main text, we describe how we use a mathematical model composed of a system of recursions to predict the equilibrium abundance of XX symbiont infected females versus the frequency of X0 feminized females. This section describes the mathematical model, analyses the equilibria of the model, and provides the frequency of XX symbiont infected females at the stable equilibrium.

We assume that the host population is large enough to be modeled by a deterministic system where stochastic effects like genetic drift are ignored. The frequencies of uninfected and infected XX females are x_u and x_i, respectively, the frequencies of uninfected and infected X0 males are y_u and y_i, respectively, and the frequency of infected and feminized female X0 individuals is z_i (see Table S1). The symbiont is transmitted successfully from mother to offspring with probability t_F, and if the offspring is male, it is feminized into a functional female with probability ϕ. Given these assumptions, we can write the frequency of each class of individual in the next generation (denoted by the $'$ symbol) as

$$\begin{aligned}
2w\, x_u' &= \left(x_u + (1-t_F)x_i + \frac{1}{2}(1-t_F)z_i\right)(y_u + y_i) \\
2w\, x_i' &= t_F \left(x_i + \frac{1}{2}z_i\right)(y_u + y_i) \\
2w\, y_u' &= (x_u + (1-t_F)x_i + (1-t_F)z_i)(y_u + y_i) \\
2w\, y_i' &= t_F(1-\phi)(x_i + z_i)(y_u + y_i) \\
2w\, z_i' &= t_F\phi(x_i + z_i)(y_u + y_i)
\end{aligned} \tag{S1}$$

where w is the mean fitness and the sum of the right hand sides of equation (S1). We normalize both the number of individuals with XX and X0 by the same mean fitness w in equation (S1) so that we can obtain a proper sex ratio.

In order to obtain the equilibrium frequencies of uninfected and infected females and males under this model, we set $x_u' = x_u = x_u^*$, $x_i' = x_i = x_i^*$, $y_u' = y_u = y_u^*$, $y_i' = y_i = y_i^*$, and $z_i' = z_i = z_i^*$ in equation (S1) and solve for the starred equilbrium values. Doing this yields three equilibria (see the Mathematica file in the Zenodo repository). The first one is the equilibrium where there are no

Variable	Genotype	Sex	Infected?
x_u	XX	female	no
x_i	XX	female	yes
y_u	X0	male	no
y_i	X0	male	yes
z_i	X0	female	yes

Table S1. Variables used in the model of feminizing symbiont spread.

infected individuals,

$$x_u^* = 1/2 \qquad x_i^* = 0 \qquad y_u^* = 1/2 \qquad y_i^* = 0 \qquad z_i^* = 0\,, \tag{S2}$$

and the second equilibrium is

$$\begin{aligned}
x_u^* &= \frac{(1-t_F)t_F\left(\phi^2 + (2+(1-3t_F)\phi)(1+\sqrt{1+\phi^2})\right)}{\phi\left(6t_F(1+\phi) - 9t_F^2\phi - 2\right)} \\
x_i^* &= \frac{t_F\left(-2\phi^2 t_F + (-2(1-t_F) + (4-3t_F)t_F\phi)(1+\sqrt{1+\phi^2})\right)}{\phi\left(6t_F(1+\phi) - 9t_F^2\phi - 2\right)} \\
y_u^* &= \frac{(1-t_F)\left(-2\phi + 2t_F(1+\sqrt{1+\phi^2}) + t_F\phi(5+5\phi-\sqrt{1+\phi^2} - 3t_F(1+\phi+\sqrt{1+\phi^2}))\right)}{\phi\left(6t_F(1+\phi) - 9t_F^2\phi - 2\right)} \\
y_i^* &= \frac{1-\phi}{\phi} z_i^* \\
z_i^* &= \frac{t_F\left(-2(1+\phi+\sqrt{1+\phi^2}) - 3t_F^2\phi(1+\phi+\sqrt{1+\phi^2}) + 2t_F(1+\sqrt{1+\phi^2} + \phi(4+\phi+\sqrt{1+\phi^2}))\right)}{6t_F(1+\phi) - 9t_F^2\phi - 2}
\end{aligned} \tag{S3}$$

and contains infected individuals. The third equilibrium is a complement to the second one above, but it is not viable (i.e., its values are not all between zero and one when $1 \leq t_f \leq 0$ and $1 \leq \phi \leq 0$).

We need to know if and when each of the first and second equilibria are stable so we next calculate the eigenvalues λ of the Jacobian matrix of the recursion in equation (S1). The Jacobian matrix (see the Mathematica file) has two zero eigenvalues and two non-zero conjugate eigenvalues

$$\begin{aligned}
\lambda_\pm = &(4x_i + 4x_u + 3z_i)^{-2} \\
&\Big(z_i + t_F(4x_i + 4x_u + 3z_i + x_i\phi + 4x_u\phi) \\
&\pm \Big((z_i + t_F(4x_i + 4x_u + 3z_i + x_i\phi + 4x_u\phi))^2 \\
&- 4t_F(4x_i + 4x_u + 3z_i)(z_i + 2x_u t_F\phi - x_i(1-2t_F)\phi)\Big)^{1/2}\Big)
\end{aligned} \tag{S4}$$

The eigenvalue λ_- is always less than one in magnitude for both the first and second equilibrium (see Mathematica file). Evaluating λ_+ shows the first equilibrium with no infected individuals is unstable and the second equilibrium is stable and exists when

$$t_F\left(1+\phi+\sqrt{1+\phi^2}\right) > 2\,. \tag{S5}$$

Thus, we should use the second equilibrium to predict our frequencies of XX versus X0 females. As a note, this is a more stringent invasion condition for the feminizing symbiont than for other models of feminization (Randerson et al., 2001; Brenninger et al., 2025) because of the cost of inviable 00 offspring produced by X0 feminized females.

The frequency of XX females among all infected females, $x_i^*/(x_i^*+z_i^*)$, using the second equilibrium is

$$-\phi + \sqrt{1+\phi^2} \tag{S6}$$

In order to use equation (S6), we still need to estimate ϕ. We can do this since we know the sex ratio or fraction of males for each of the symbiotypes. The equilibrium fraction of males assuming that $t_f = 1$ is

$$y_u^* + y_i^* = \frac{(1-\phi)(3-\phi-\sqrt{1+\phi^2})}{4-3\phi} \tag{S7}$$

Inserting the empirical fraction of males from each symbiotype into equation (S7), we can estimate ϕ by solving equation (S7) and then estimate the fraction of infected females who are XX for each symbiotype using equation (S6) (see data and Mathematica file in the Zenodo repository).

2 PREDICTED DISTRIBUTION OF EGG MORTALITY

In Section 1, we derive a method to estimate the fraction of XX and X0 infected at equilibrium. Now, we use this information to predict the mortality or inviablity fraction of eggs in eggmasses of infected females. We assume that there is some baseline mortality or inviability probability of s for all eggs. If an infected females is XX, we assume this is the only source of mortality for her eggs. Assuming that the survival of each egg in the eggmass is independent and the number of eggs in the eggmass is n, the number of surviving eggs K_{X} of an infected XX female follows a binomial distribution with parameters n and s or $K_{\mathrm{X}} \sim \mathrm{Binomial}(n, s)$. If an infected female is X0, 1/4 of her offspring will be inviable 00 individuals, and her number of surviving offspring K_0 in an eggmass of size n will be $K_0 \sim \mathrm{Binomial}(n, 3s/4)$. If the fraction of XX females among all infected females is x, the number of surviving offspring K of a random female is a random variable with a probability density function that is a mixture of the two binomial distributions Binomial(n, s) and Binomial$(n, 3s/4)$:

$$x \binom{n}{k} s^k (1-s)^{n-k} + (1-x) \binom{n}{k} (3s/4)^k (1-3s/4)^{n-k} \, . \tag{S8}$$

For each eggmass in each symbiotype in the empirical data, we use equation (S8) to generate a random number K of surviving eggs for that eggmass that is specific to the size n of that eggmass. Averaging over all the empirical eggmasses for a symbiotype generates a random survival fraction (and mortality or invability fraction) for each symbiotype. We generate 500,000 such random mortality fractions to estimate the predicted mortality distribution for each symbiotype. The estimated symbiotype-specific mortality distributions are used to calculate the 95% confidence intervals in Figure 3A in the main text (see Mathematica file for reproducible code).

REFERENCES

Brenninger, F. A., Zug, R., and Kokko, H. (2025). Infection dynamics of endosymbionts that manipulate arthropod reproduction. Biological Reviews 100, 1787–1812. doi:10.1111/brv.70024

Randerson, J., Moreau, J., Rigaud, T., and Hurst, L. D. (2001). Understanding the distribution and effects of Wolbachia: the co-existence of cytoplasmic incompatibility and feminization. Selection 2, 237–248. doi:10.1556/select.2.2001.1-2.17
